# The Physicians Visiting List for 1891

**Published:** 1891-01

**Authors:** 


THE PHYSICIANS' VISITING LIST for 1891. P. Blakiston, Son & Co., Publishers, Philadelphia. This valuable visiting list has reached the fortieth year of its publication. It consists of Regular Interleaves, and Perpetual Editions. Its contents comprises Almanac; Table of Signs; Marshall Hall's ready method in Asphyxia; Poisons and Antidotes; Metric or French Decimal System of Weights and Measures; Dose Table, List of New Remedies; Aids to Diagnosis and Treatment of Diseases; Diagram of Eruption of Deciduous Teeth, Posological Table; Disinfectants; Examination of Urine; Incompatibility; New Table for Calculating the period of Utero-gestation; Sylvester's Method of Resuscitation; Transportation of Injured Persons and Diagram of the Chest; all of which render it a most useful and convenient list. It also proves a valuable and convenient appointment list for the dental practitioner.

